# Chronic activation of inflammasome signaling complexes and enhancement of behavioral abnormalities

**DOI:** 10.1192/j.eurpsy.2023.731

**Published:** 2023-07-19

**Authors:** B. Abdelmoula, N. Bouayed Abdelmoula

**Affiliations:** Genomics of Signalopathies at the service of Medicine, Medical University of Sfax, Sfax, Tunisia

## Abstract

**Introduction:**

Inflammasomes are cytosolic multi-component signaling platforms critical to the innate immune response to infectious diseases and the dysregulation of their activation can lead to the development of neurodegeneration and cancer.

**Objectives:**

We aim through this review to ass a possible interplay between dysregulation of inflammasome activation, development of chronic inflammatory disease and enhancement of behavioral abnormalities.

**Methods:**

We comprehensively review the scientific literature using Pubmed database and other search platforms such as Google scholar to assess the role and the actors of chronic activation of inflammasome signaling complexes to establish a potential association between dysregulation of inflammasome activation, chronic inflammatory disease and enhancement of behavioral abnormalities.

**Results:**

Our bibliographic review revealed that dysregulation of the inflammasome is associated with the onset and progression of several autoinflammatory and autoimmune diseases, including cryopyrin-associated periodic fever syndrome, familial Mediterranean fever, rheumatoid arthritis, and systemic lupus erythematosus. These multimeric complexes form in response to molecular patterns unique to pathogens and cellular damage, triggering a cascade of downstream responses, including the induction of pyroptotic cell death and release of proinflammatory cytokines. Some inflammasomes directly recognize these patterns, while others indirectly sense these patterns through changes in the homeostatic environment of the cell. Moreover, although being a normal part of the skin flora, yeasts of the genus Malassezia are associated with several inflammatory skin diseases including pityriasis versicolor (tinea versicolor), atopic eczema, psoriasis, Malassezia folliculitis and onychomycoses. In the context of tolerating fungi during colonization and eliciting, activation, of inflammasomes signaling complexes, has been identified as an integral part of antifungal host defense. While the activation of inflammasomes mainly the NLRP3 one, was shown to be pivotal for innate immunity against pathogenic fungi such as candida albicans, their role in the fungal genus Malassezia remains imprecise. Even though, many observations suggest that simultaneous activation of NLRP3, NLRC4 and AIM2 inflammasomes may play an important role.

**Conclusions:**

Whereas, chronic inflammasome activation such as by chronic infectious has been tied to the development of metabolic syndromes, neurodegenerative diseases, and cancer progression, a possible interplay between chronic invasion by the genus Malassezia, vigorous immune response to eliminate invading fungal pathogens, disruption of immune sensors of genotoxic stress, development of chronic inflammatory disease and behavioral abnormalities may be a new field of scientific researches.

**Disclosure of Interest:**

None Declared

